# Regional Decline of Coral Cover in the Indo-Pacific: Timing, Extent, and Subregional Comparisons

**DOI:** 10.1371/journal.pone.0000711

**Published:** 2007-08-08

**Authors:** John F. Bruno, Elizabeth R. Selig

**Affiliations:** 1 Department of Marine Sciences, The University of North Carolina at Chapel Hill, Chapel Hill, North Carolina, United States of America; 2 Curriculum in Ecology and Department of Marine Sciences, The University of North Carolina at Chapel Hill, Chapel Hill, North Carolina, United States of America; University of Sheffield, United Kingdom

## Abstract

**Background:**

A number of factors have recently caused mass coral mortality events in all of the world's tropical oceans. However, little is known about the timing, rate or spatial variability of the loss of reef-building corals, especially in the Indo-Pacific, which contains 75% of the world's coral reefs.

**Methodology/Principle Findings:**

We compiled and analyzed a coral cover database of 6001 quantitative surveys of 2667 Indo-Pacific coral reefs performed between 1968 and 2004. Surveys conducted during 2003 indicated that coral cover averaged only 22.1% (95% CI: 20.7, 23.4) and just 7 of 390 reefs surveyed that year had coral cover >60%. Estimated yearly coral cover loss based on annually pooled survey data was approximately 1% over the last twenty years and 2% between 1997 and 2003 (or 3,168 km^2^ per year). The annual loss based on repeated measures regression analysis of a subset of reefs that were monitored for multiple years from 1997 to 2004 was 0.72 % (n = 476 reefs, 95% CI: 0.36, 1.08).

**Conclusions/Significance:**

The rate and extent of coral loss in the Indo-Pacific are greater than expected. Coral cover was also surprisingly uniform among subregions and declined decades earlier than previously assumed, even on some of the Pacific's most intensely managed reefs. These results have significant implications for policy makers and resource managers as they search for successful models to reverse coral loss.

## Introduction

There is growing scientific and public awareness of the widespread depletion of marine habitat-forming species, such as mangroves, seagrasses, oysters, and corals [e.g., 1,2,3]. This loss inevitably leads to the decline of the plants and animals that live in the biogenic structures created by such foundation species, and contributes to the overall degradation of marine ecosystems [4]. For example, the reduction of coral cover on tropical coral reefs directly and rapidly causes a decline in the abundance and diversity of reef fish through the loss of structural heterogeneity [5], [6].

**Figure 1:**
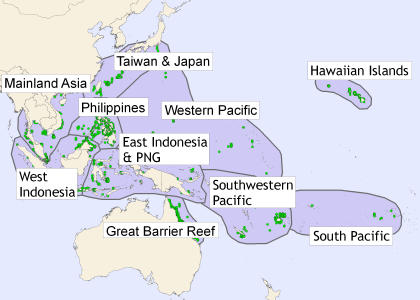
Map of study region, sub-regions, and the 2667 surveyed reefs (green dots).

Scientists have recognized the ecological and economic value of coral reefs and the threats to reef-building corals for decades [7]-[9] and there is broad scientific consensus that coral reef ecosystems are being rapidly degraded [10], [11]. Yet there is little published empirical information on regional and global patterns of coral loss [12] or the current state of reefs in the Indo-Pacific (Fig. 1)[13]. This region encompasses approximately 75% of the world's coral reefs (Text S1) and includes the center of global marine diversity for several major taxa including corals, fish, and crustaceans [14]. Many previous studies have documented mass coral mortality events and ecologically significant reductions in coral cover on particular reefs [15]-[19], throughout the Caribbean [12], and across the Great Barrier Reef [20], [21]. However, the inference that this decline is a general, global phenomenon is based largely on qualitative assessments [e.g., 22,23]. The absence of regional-scale quantitative analyses of reef health in general and coral cover in particular has led to substantial confusion and disagreement about the patterns and causes of coral decline [24], [25]. This shortcoming has also greatly limited our ability to measure the efficacy of different management practices designed to mitigate and reverse reef degradation [12], [26].

Here we describe a comprehensive analysis of the timing, rate, and geographic extent of the loss of coral cover across the Indo-Pacific (Fig. 1). For the purposes of this study, the Indo-Pacific region is defined by the Indonesian island of Sumatra in the west (95°E) and by French Polynesia in the east (145.5°W) (Fig. 1). We compiled a coral cover database that included 6001 quantitative surveys of 2667 subtidal coral reefs (Fig. 1, Map S1, Tables S1 and S2, Text S2) performed between 1968 and 2004. The surveys were performed by scientists or trained volunteers using either *in situ* or photographic/video-based measurements. Because corals facilitate so many reef inhabitants [5], [6], [27], living coral cover is a key measure of reef habitat quality and quantity, analogous to the coverage of trees as a measure of tropical forest loss.

This study provides the first regional scale and long-term analysis of coral cover in the Indo-Pacific. Our results indicate that the loss of coral cover began earlier than assumed and that coral cover is currently very similar across the Indo-Pacific, suggesting that coral decline is a general global phenomenon.

## Methods

### Data sources

Our analyses were based on quantitative surveys that measured the percentage of the bottom covered by living scleractinian corals on subtidal coral reefs (1–15 m depth, mean survey depth was 6.2 m) within ten subregions of the Indo-Pacific (Fig. 1, Table S1). We included data from several sources including the published results of academic, governmental, and non-governmental organization (NGO) scientists and, for one source (Reef Check), volunteers trained and supervised by professional scientists (Table S2). We used a number of online literature search tools (e.g., ISI Web of Science and Google Scholar) to find published peer review and gray literature sources of coral cover data (Text S2) using search terms including “coral” and “cover”, “reef” and “health”. We also browsed all available issues of several relevant journals including Atoll Research Bulletin, Coral Reefs, Marine Pollution Bulletin, and the Proceedings of the International Coral Reef Symposium and similar regional symposia on reef ecology and conservation. All of the data collected before 1984 are from such published sources or ReefBase.

Coral cover data from the Australian Institute of Marine Science's (AIMS) Long Term Monitoring Program (LTMP) [28] were available for 1986–2004 and NGOs only began intensive reef monitoring a decade ago (Table S2). Therefore, there is a shift in the dominant data sources over time as well as a standardization of survey techniques. All reef monitoring databases that we obtained coral cover data from are publicly accessible. Portions of the coral cover data from these sources including AIMS, the National Oceanic and Atmospheric Administration (NOAA), the Hawaii Coral Reef Assessment and Monitoring Program (CRAMP), and Reef Check (Table S2) have been published independently [29], [20], [28], [30]. However, they have not previously been analyzed collectively or combined with other data sources or published as a comprehensive evaluation of regional coral cover change and status.

Most surveys were based on the line transect technique or some variant to estimate coral cover. A transect (typically 10–30 m in length and usually a tape measure or chain) is placed on the reef, oriented either along a depth contour or down the reef slope. Coral cover is then estimated either *in situ* by recording the number of points along each transect (at either set intervals or random locations) that overlay a living hard coral (the point intercept technique) or by taking digital or film images of the bottom at these points within quadrats (usually 0.25 m^2^ or 1.0 m^2^). In most surveys, multiple transects or quadrats were used to estimate cover at a given reef, producing more than one cover value. We always pooled such replicate cover measurements from one depth/zone into a single mean estimate.

We also included data from manta tow surveys of coral cover [31] performed by the AIMS LTMP (1231 surveys performed at 136 reefs between 1986 and 2003). AIMS uses extensive training [32] and quality control procedures [31] to verify the validity of their manta tow surveys, which are unbiased and highly comparable to surveys based on video transects [33]. The manta tow technique is frequently used to perform broadscale reef surveys [e.g., 34]. Because we were not able to verify the validity and reproducibility of manta tow surveys performed by other organizations, we only included AIMS manta tow data in our analyses.

### Reef monitoring data

Most of the reefs in the database were surveyed only once, but a subset of 651 reefs were surveyed two or more times (Table S3). In most cases, transects or manta tow paths on these monitoring sites were permanently marked or recorded using GPS so that the exact same location on each reef could be resurveyed in subsequent years. There are more monitoring sites on the GBR than within other subregions, especially from 1984 to 1996. However, 67% of the monitoring sites were within the other nine subregions, and some other subregions including the Philippines and mainland Asia also had a relatively large number of monitoring sites (Table S3).

### Statistical analysis

Linear repeated measures regression analysis was used to test the null hypothesis that there was no relationship between coral cover and time from 1968 to 2004. We were unable to perform a formal meta-analysis because several critical components (e.g., variance estimates, sample size, repeated sampling of each reef, etc.) were not available for all data sets. We used Stata (version 9.1, STATA Corp.) and performed two sets of analyses: (1) on the annual subregional means based on all 6001 surveys, and (2) on the data from the 651 monitoring sites. In both analyses, time (year) and coral cover were treated as continuous variables. Because locations were repeatedly sampled over time, coral cover estimates of a given subregion or reef in different years were not independent. This longitudinal structure was incorporated into the statistical model by using repeated measures of subregions or reefs. Thus, statistical estimates of the absolute net decline in coral cover were based on the individual trajectories of subregions or reefs and were not derived by pooling all the data for each year. For these and all other analyses, data were transformed when necessary to meet basic statistical assumptions.

In the subregion analysis, we used the mean cover in each subregion for each year as the dependent variable, rather than the individual reef means, in part because the sample size varied greatly among years, periods, and subregions. Performing this analysis on yearly subregional averages equalizes the influence of each subregion and prevents the results from being driven primarily by especially well-sampled subregions like the GBR and the Philippines (Table S3). However, this procedure did not remove the influence of either intentionally or unintentionally biased sampling within subregions that could have caused the estimated coral cover means to differ from the true subregional population means.

For the analysis of the reef monitoring data, we performed four repeated measures regression analyses to test the null hypothesis and estimate the slope of significant linear functions during the entire 36 year range and for each of the three periods: 1970–1983, 1984–1996, and 1997–2004. The period delineations were based on the timing of major disturbance events and expected and observed trends in coral cover in the Indo-Pacific. For example, the beginning of the third period (1997–2004) coincides with a major global mass-bleaching event in 1998 and 1999 [18], [35], fairly rapid declines in coral cover in several subregions between 1996 and 1998 (Fig. S1), and the beginning of an eight year decline of mean regional coral cover. We also repeated the analysis of the last two time periods without the GBR monitoring sites to assess their influence.

Estimates of the rate of coral loss could be influenced by year-to-year and period-to-period changes in the location of reef surveys. For example, if surveys initially focused on high cover reefs or subregions and then shifted focus to low cover reefs, the estimated rate of regional or subregional coral loss could be exaggerated. Alternatively, an initial overrepresentation of low cover reefs or subregions could underestimate the true rate of net coral loss. This problem is diminished in the monitoring sites analysis because individual reefs are monitored through time and reef identity is far less variable. Nevertheless, the identity of monitored reefs did change over time (e.g., when new reefs and subregions were added), so this potential source of bias was not entirely eliminated. A second potential bias in the analyses is the overrepresentation of the best-sampled subregions, mainly the Philippines and the GBR. Therefore, the regression results are not necessarily representative of all ten subregions, especially those that were not well monitored.

Because the effects of a variety of disturbances on coral cover are depth-dependent [36]–[38], [18], spatial and temporal variability of the depth of reef surveys could complicate our analyses. Therefore we conducted an extensive analysis of the potential confounding effects of depth on our subregional comparisons and rate estimations (Text S3, Table S4).

## Results and Discussion

**Figure 2:**
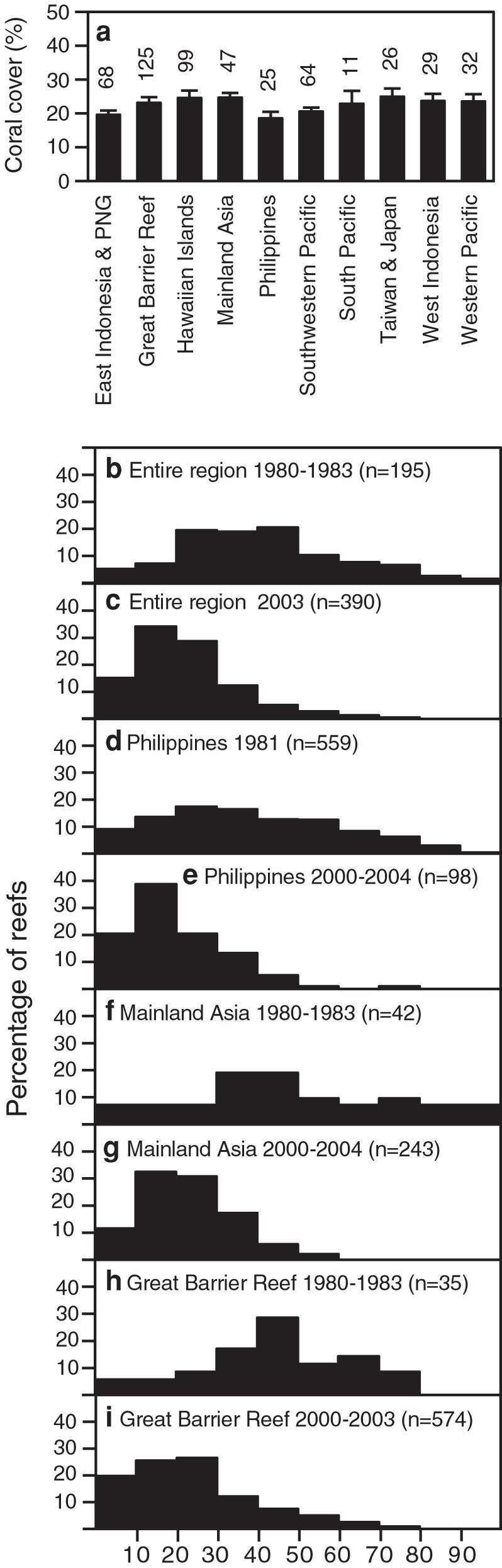
Coral cover in the Indo-Pacific. (a) Cover (means ± 1 SE) in ten subregions of the Indo-Pacific. Data are from 2003 for seven subregions and from 2002 for three subregions not adequately sampled after 2002 (Hawaiian Islands, Taiwan & Japan, and Western Pacific). Values above the bars are the number of reefs surveyed in each subregion. (b-i) Histograms illustrating percent coral cover in the Indo-Pacific and selected subregions during different periods. (d) is based on [45].

Our results indicate that coral cover on Indo-Pacific reefs is currently lower and far more uniform than expected (Fig. 2A). The region-wide average was only 22.1% in 2003 (95% CI: 20.7, 23.4, n = 390 reefs) and did not vary significantly among subregions (Kruskal-Wallis p = 0.19, ANOVA p = 0.40, Power = 0.91 when δ = 3)(Fig. 2A). A number of factors thought to influence coral reef resilience including management resources and enforcement, coral diversity, and human population density and social structure, vary substantially among the ten subregions [13], [14]. Therefore, the observed uniformity of the average coral cover in 2003 across the entire region is one of the most surprising results of our analysis.

The general absence of quantitative data on reef health has led to several misconceptions about the causes, patterns, and best remedies for global coral decline. For example, in 2003, coral cover on the Great Barrier Reef (GBR), considered the “best-managed” [25] and “one of the most ‘pristine’ coral reefs in the world” [21], was not significantly greater than on reefs in the Philippines and other subregions that are often thought to be highly threatened and poorly managed [13]. Additionally, based on the impression that Hawaiian reefs were “far further down the trajectory of decline” [than reefs in the Caribbean and Australia] a recent essay [25] argued for a total overhaul of U.S. coral reef management policy. But our analysis suggests that coral cover in the main Hawaiian islands, including frequently visited reefs close to urban and tourism centers, appears to have been as high as GBR cover over the last two decades (Fig. 2A; also see Fig. S5). However, we necessarily combined data from surveys using different techniques and protocols for site selection which could have exaggerated or obscured differences in coral cover among subregions (Text S4).

Additionally, there are other important measures of reef degradation, in particular the abundance and diversity of reef inhabitants [23]. It is possible that there is currently greater variance in metrics such as fish biomass among subregions of the Indo-Pacific. However, for these metrics there is far less available information, substantial natural variance across the region due to differences in local productivity and reef connectivity, and greater uncertainty about historical baselines. Thus direct geographic comparisons would be more difficult. The focus of this investigation was coral cover; given the key role that corals play in facilitating the entire reef ecosystem, coral cover is a critical measure of habitat loss and degradation. Nonetheless, there is an urgent need for similar regional-scale comparative studies of the health of populations of commercially and ecologically important reef inhabitants.

Historically, i.e., 100–1000 y.b.p., average coral cover in the Indo-Pacific was probably approximately 50% [39]. Generating natural baselines to estimate the long-term impact of human activities on species and ecosystems is always difficult, particularly in the ocean since records are rarely kept until a resource is already significantly depleted [40]. Humans have affected fringing reefs close to inhabited islands for hundreds of years via overfishing and land use practices that lead to increased sedimentation [41], [23]. But these effects were localized and it is doubtful that humans significantly influenced regional or subregional average coral cover before the twentieth century. Some of the earliest quantitative Indo-Pacific reef surveys reported local coral cover of nearly 90% [42]. However, it is unlikely that such values represented regional or even subregional averages, even when the Indo-Pacific was pristine. Coral reefs have always been affected by a variety of natural disturbances including severe storms that can drastically reduce coral cover [43], [7], [15]. Even in a pristine, pre-human state, some proportion of reefs within a subregion would be in a state of recovery from a recent disturbance, thereby reducing subregional coral cover averages.

**Figure 3:**
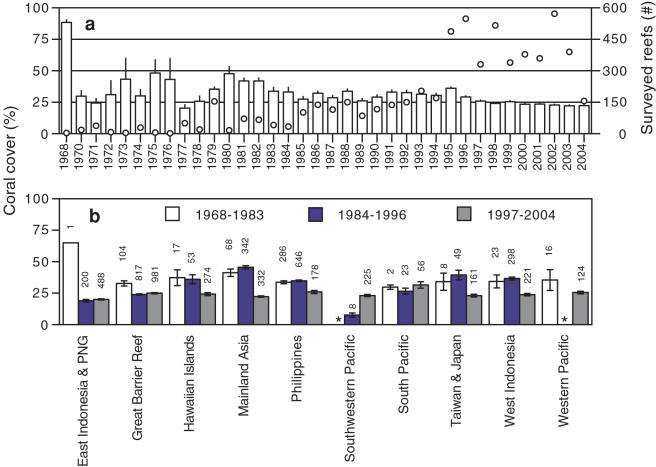
Regional trends in coral cover between 1968 and 2004. (a) White bars represent Indo-Pacific coral cover and open symbols (right axis) are the number of reefs surveyed each year. (b) Coral cover in ten Indo-Pacific subregions in each of three periods. Plotted values are means±1 SE and values above each bar are the subregional sample sizes. * = no data were available

We may never know the precise Indo-Pacific coral cover baseline, but we now know that regionally, cover is currently at least 20% below the best historical reference points. Our results suggest that average Indo-Pacific coral cover declined from 42.5% during the early 1980s (95% CI: 39.3, 45.6, n = 154 reefs surveyed between 1980 and 1982) to 22.1% by 2003 (Fig. 3A); an average annual cover loss of approximately 1% or 1,500 km^2.^ However, coral cover fluctuated somewhat throughout the 1980s and the regional average was still 36.1% in 1995 (95% CI: 34.2, 38.0, n = 487), subsequently declining by 14% in just seven years (or 3,168 km^2^ year^−1^). We used repeated measures regression analysis based on the individual trajectories of subregions or reefs (for the analysis of the reef monitoring data) to quantitatively estimate the absolute net decline of coral cover. Estimates based on subregional means and the reef monitoring data (a subset of the entire database) for similar periods were nearly identical (Table 1) and were slightly lower than estimates based on annual pooling (described above).

**Table 1:** Results of linear repeated measures regression analyses on the relationship between coral cover and time in the Indo-Pacific.

Analysis	n	df	F	p	R^2^	Slope (95% CI)
Subregional means (1968–2004)	213	1,9	9.06	0.015	0.05	−0.37 (−0.64, −0.09)
Monitoring sites (1970–2004)	2994	1, 651	25.94	<0.0001	0.02	−0.39 (−0.54, −0.24)
Monitoring sites (1970–1983)	186	1,109	16.07	<0.0001	0.09	1.53 (0.77, 2.29)
Monitoring sites (1984–1996)	1299	1,395	0.95	0.33	0.0013	−0.19 (−0.57, 0.19)
Monitoring sites (1997–2004)	1509	1,475	15.24	<0.0001	0.01	−0.72 (−1.08, −0.36)
Monitoring sites except GBR (1984–1996)	502	1,210	0.77	0.383	0.0027	−0.29 (−0.93, 0.36)
Monitoring sites except GBR (1997–2004)	594	1,280	7.75	0.006	0.0162	−0.90 (−1.53, −0.26)

Analyses were based on the individual, independent trajectories of subregions or reefs (for the monitoring sites analyses). n = total number of observations

The estimated annual rate of coral cover loss in the Caribbean between 1977 and 2001 was approximately 1.5%, with the greatest decline occurring during the 1980s [12]. In contrast, the estimated net annual loss of global humid tropical rainforest was only 0.4 % from 1990–1997 [44]. Additionally, the spatial patterns of coral reef degradation are very different from rainforest loss in that nearly all reefs have been affected; there are virtually no remaining pristine reefs and very few with coral cover close to the historical average (Fig. 2C). Remarkably, in 2003, only 4% of the 390 surveyed Indo-Pacific reefs had coral cover >50% and only 2% had cover >60%. In contrast, cover was ≥50% on nearly a third of the reefs surveyed between 1980 and 1983 (Fig. 2B). This striking shift in the distribution of coral cover is apparent in several subregions (Figs. 2B–H). For example, a landmark study of Philippine reef health in 1981 [45] found that coral cover was greater than 25% on 68% of 559 surveyed reefs. Twenty years later, only 26% of reefs in the Philippines had cover greater than 25% (Figs. 2D & E).

Regional and subregional trends in coral cover during the 1970s are less clear than for more recent periods because fewer surveys were performed, few subregions were adequately sampled, and 77% of surveyed reefs prior to 1973 were on the GBR. Therefore, it is unlikely that the regional cover average of 30% between 1968 and 1972 (95% CI: 23.5, 35.5, n = 70) is representative of all subregions, particularly those that did not experience outbreaks of *Acanthaster plancii*, a corallivorous sea star that substantially reduced cover on many GBR reefs [36]. *Acanthaster* predation had similar effects in Guam, Fiji, Palau and other locations over the last forty years and is a principle cause of coral loss in several subregions [46], [47]. But many Indo-Pacific reefs affected by *Acanthaster* in the 1960s and 1970s partially or wholly recovered by the early 1980s [48], [15], [47]. Our analysis of coral cover data from the 651 reef monitoring sites reflects this recovery (Table 1). Cover on 110 reefs monitored between 1970 and 1983 increased significantly, which is concordant with documented cases of local increases in coral cover following major disturbances prior to this period [e.g., 48,47]. Coral cover on the reef monitoring sites did not change significantly between 1984 and 1996 but has declined substantially since 1997 (0.72% per year, 95% CI: 0.36, 1.08).

Our analysis suggests that the regional-scale coral decline in the Indo-Pacific began several decades earlier than often assumed. For example, Pandolfi et al. [23] and others [21], [25] have argued that due to greater coral diversity, superior management practices, and a variety of historical socio-economic factors, coral cover on Indo-Pacific reefs in general and on the GBR in particular declined much more recently than in the Caribbean. However, our results indicate Indo-Pacific coral cover was already quite low, and in some subregions substantially declining during the 1960s and 1970s (Fig. 3 and Fig. S1). This finding is consistent with often overlooked published studies over the last forty years that documented localized coral decline in the Indo-Pacific, particularly after *Acanthaster* outbreaks. For example, Endean and Stablum [36] quantified the collapse of coral cover on 19 reefs on the GBR to 16.8±5.6 % (mean±1 SE) by 1970, fifteen years before similar broad scale coral mortality was observed in the Caribbean [16], [12].

Comparing the timing and rate of coral decline among Indo-Pacific subregions is difficult because many were not adequately sampled until the early 1980s. Furthermore, historic baseline coral cover may have varied among subregions due to differences in disturbance frequency or the morphology of dominant species. For example, reefs dominated by plating acroporiid corals probably had higher baseline cover than reefs dominated by branching corals. Thus, similar current cover among subregions could actually reflect variability in the degree of coral loss. Additionally, the dependence of facilitation and other ecosystem functions on coral cover could vary among subregions (e.g., 20% cover might not be universally functionally equivalent).

Between 1984 and 1996, coral cover was slightly lower in east Indonesia than on the GBR (Fig. 3B). However, cover in several other subregions was substantially higher during this period, particularly in mainland Asia, Taiwan and Japan, and west Indonesia (Fig. 3B & S2). Absolute coral cover in these subregions declined by 10–20% between 1996 and 1998, possibly due in part to El Niño-related bleaching [49], [18]. Well-documented mass coral bleaching events driven by elevated seawater temperatures have caused coral mortality throughout the Indo-Pacific, particularly in 1998, 1999, and 2002 [50], [51], [18], [35].

Major storms, though not novel disturbances, are considered primary causes of recent coral loss in several locations including Hawaii and Moorea [52], [7]. Infectious coral disease epidemics are not thought to be as prevalent or important in the Pacific as they are in the Caribbean [53], although this could be largely due to limited Indo-Pacific disease research [54]. In fact, recent surveys of coral diseases on the GBR indicate that disease frequency increased dramatically over the last five years, particularly on the outer GBR [54] and following periods when water temperature was anomalously high [55], [56]. In addition to these regional-scale stressors, more localized human impacts have also caused coral losses. Examples include sedimentation from urban development and agriculture in Hong Kong and Papua New Guinea, respectively [13], [57], [5], and destructive fishing practices such as blast fishing and muro-ami (a form of destructive net fishing) in Indonesia and the Philippines [58].

Despite the well-documented effects of several causes of mass coral mortality, there is substantial evidence that coral communities remain resilient, often recovering in ten to thirty years after major disturbances [15], [47], [20], [39], [59]. However, such “recovery,” loosely defined as a return to pre-disturbance coral cover, often does not mean a return to original coral species composition because the recovery of slow-growing species can take centuries. Such compositional shifts can influence reef geomorphology, the structure of associated invertebrate and fish communities, and resilience to future disturbances.

**Figure 4:**
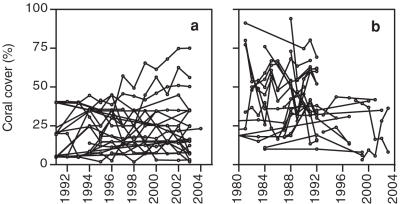
Illustrative examples of asynchrony of coral cover among 25 randomly selected monitored reefs on the GBR (a) and in Indonesia (b).

Average GBR coral cover has been consistently below 27% since 1986 (Fig. S1). But this subregional stability, also apparent in most other subregions over the last ten years (Fig. S1), masks complex within-subregion dynamics (Figs. 4A & B)[20]. The cover and trajectories of individual reefs, in many cases separated by only a few kilometers, remains surprisingly unpredictable. This small scale spatial asynchrony in coral cover is likely caused in part by the highly localized effects of even regional scale disturbances, including predator and disease outbreaks and thermal anomalies that cause coral bleaching [35], [55]. The frequency and spatial variability of these disturbances have prevented recovery at subregional and regional scales despite significant local increases in coral cover on many reefs. Such asynchrony also increases intra-annual variability, reducing the amount of variance explained by time in the regression analyses.

### Conclusions

The results of our analysis of 6001 quantitative reef surveys indicate that the degree, geographic extent, and duration of the Indo-Pacific coral decline have been significantly underestimated. Many coral reef scientists know of exceptions to the general pattern of reef degradation: there are currently many, perhaps hundreds or even thousands of high coral cover (i.e., >60%) reefs in the Indo-Pacific and Caribbean that resemble the presumed historical coral baseline [e.g.,59]. But our results indicate that such observations are anomalies and currently represent less than 2% of reefs in the Indo-Pacific. This study also highlights the urgent need for conservation policies to restore coral reefs and the ecosystem services they provide, estimated to be worth $23,100–$270,000 km^−2^ year^−1^ [13]. Halting and reversing coral loss will require actions across a range of scales including local restoration and conservation of herbivores that facilitate coral recruitment [60], [61] and the reduction of fishing practices that directly kill corals [58], the implementation of regional land use practices that reduce sedimentation and nutrient pollution [57], and the institution of global policies to reduce anthropogenic ocean warming and acidification [62], [11].

The loss of coral cover represents both an absolute loss and a reduction in the quality of reef habitat [63]. Coral reefs, like nearly all aquatic and terrestrial habitats, are hierarchically organized and are wholly dependent on the presence of the foundation species that generate the physical reef framework [4]. Ecosystem management should also be hierarchical and begin with the preservation of foundation species. Unfortunately, most marine conservation policies focus on the commercially harvested occupants of these habitats. Such remedies will fail unless we gather the scientific knowledge and political will needed to effectively reduce the stressors degrading corals and other marine foundation species.

## Supporting Information

Text S1 Calculation of Indo-Pacific reef area
(0.03 MB DOC)

Text S2 Published data sources
(0.04 MB DOC)

Text S3 Analysis of potential effects of depth on coral cover estimates
(0.05 MB DOC)

Text S4 Potential biases in survey techniques and site selection
(0.05 MB DOC)

Table S1 The number of surveyed reefs in the ten Indo-Pacific subregions.
(0.05 MB DOC)

Table S2 Characteristics of the eight basic sources of coral cover data.
(0.05 MB DOC)

Table S3 Number of monitoring sites in the ten Indo-Pacific subregions during each of three periods (note most monitoring sites were surveyed for more than one period).
(0.05 MB DOC)

Table S4 Results of linear repeated measures regression analyses on the relationship between coral cover and time in the Indo-Pacific. Unlike the results presented in Table 1, these analyses include survey depth as a covariate. The effect of depth was non-significant (Subregional analysis p = 0.90, Monitoring sites p = 0.60). Results presented in the table are for the time effect. n = total number of observations
(0.04 MB DOC)

Figure S1 Patterns of coral cover decline in ten Indo-Pacific subregions. Black bars are mean coral cover ± 1 SE for each year (missing bars are years in which no data are available). Open symbols (right axis) are the number reefs surveyed in each subregion during each year (note changes in scale).
(0.73 MB EPS)

Map S1 Locations of the 2667 surveyed reefs (green dots). (This KML file can be viewed with the Google Earth mapping system.)
(0.09 MB ZIP)
