# Supplementary material for: Regional Decline of Coral Cover in the Indo-Pacific: Timing, Extent, and Subregional Comparisons
Source: PLoS One. 2007 Aug 8;2(8):e711. doi: 10.1371/journal.pone.0000711 (PMC1933595; doi:10.1371/journal.pone.0000711)
Supplement: Text S1 — Calculation of Indo-Pacific reef area (0.03 MB DOC) [file pone.0000711.s001.doc]

**Text S1: Calculation of Indo-Pacific reef area**

We estimated that the total coral reef area in the Indo-Pacific (as defined in Fig. 1) was 158,394 km^2^ and that global reef area was 212,579 km^2^. These estimates are similar to other recent, GIS-based estimates of regional and global coral reef coverage [e.g.,1]. Our global reef area estimate is lower likely due to poorer data quality in the Indian Ocean and the Eastern and Central Pacific. We began with a GIS layer developed from Reefs at Risk in Southeast Asia [2], which used data from the United Nations Environment Program – World Conservation Monitoring Centre (UNEP-WCMC). These data were then improved with data from local experts, governmental agencies, and scientists. We added GIS data from ReefBase and Reef Check to capture reef areas in the Central Pacific. Data were both polygons and points and were gridded at a resolution of 1 km.

**Literature Cited**

1. Spalding MD, Grenfell AM (1997) New estimates of global and regional coral reef areas. Coral Reefs 16: 225-230.

2. Burke L, Selig E, Spalding M (2002) Reefs at risk in Southeast Asia. Washington, DC: World Resources Institute. 72 p.
