# Supplementary material for: Regional Decline of Coral Cover in the Indo-Pacific: Timing, Extent, and Subregional Comparisons
Source: PLoS One. 2007 Aug 8;2(8):e711. doi: 10.1371/journal.pone.0000711 (PMC1933595; doi:10.1371/journal.pone.0000711)
Supplement: Text S2 — Published data sources (0.04 MB DOC) [file pone.0000711.s002.doc]

**Text S2: Published data sources**

This reference list includes published sources we acquired for this analysis but not sources that were included in other databases (i.e., from ReefBase and Reefs at Risk in Southeast Asia) that were incorporated into our larger database.

Adjeroud, M. 1997. Factors influencing spatial patterns on coral reefs around Moorea, French Polynesia. Marine Ecology-Progress Series 159:105-119.

Adjeroud, M., D. Augustin, R. Galzin, and B. Salvat. 2002. Natural disturbances and interannual variability of coral reef communities on the outer slope of Tiahura (Moorea, French Polynesia): 1991 to 1997. Marine Ecology-Progress Series 237:121-131.

Alino, P. M., P. V. Banzon, H. T. Yap, E. D. Gomez, J. T. Morales, and R. P. Batometo. 1985. Recovery and recolonization on a damaged backreef area at Cangaluyan Is. (Northern Philippines). Proceedings of the Fifth International Coral Reef Congress 4:279-284.

Booth, D. J., and G. A. Beretta. 2002. Changes in a fish assemblage after a coral bleaching event. Marine Ecology-Progress Series 245:205-212.

Bouchon, C. 1985. Quantitative study of scleractinian coral communities of Tiahura reef (Moorea Island, French Polynesia). Proceedings of the Fifth International Coral Reef Congress 6:279-284.

Brown, B. E., M. D. Le Tissier, R. P. Dunne, and T. P. Scoffin. 1993. Natural and anthropogenic disturbance on intertidal reefs of S.E. Phuket, Thailand 1979-1992. Pages 279-285 *in* R. N. Ginsburg, editor. Proceedings of the Colloquium on Global Aspects of Coral Reefs: Health, Hazards and History. University of Miami, Miami.

Bruno, J. F., C. E. Siddon, J. D. Witman, P. L. Colin, and M. A. Toscano. 2001. El Nino related coral bleaching in Palau, Western Caroline Islands. Coral Reefs 20:127-136.

Burke, L., E. R. Selig, and M. D. Spalding. 2002. Reefs at Risk in Southeast Asia. World Resources Institute, Washington, DC.

Chansang, H., and N. Phongsuwan. 1993. Health of fringing reefs of Asia through a decade of change: a case history from Phuket Island, Thailand. Pages 286-292 *in* R. N. Ginsburg, editor. Proceedings of the Colloquium on Global Aspects of Coral Reefs: Health, Hazards and History. University of Miami, Miami.

Chou, L. M., V. S. Tuan, PhilReefs, T. Yeemin, A. Cabanban, Suharsono, and I. Kessna. 2002. Status of Southeast Asia coral reefs. Pages 123-152 *in* C. Wilkinson, editor. Status of coral reefs of the world: 2002. Australian Institute of Marine Science, Townsville.

Clark, T. H. 1996. The distribution of hermatypic Scleractinian corals at Cape d'Aguilar, Hong Kong. Pages 151-164 *in* B. Morton, editor. Proceedings of the Third International Conference on the Marine Biology of the South China Sea. Hong Kong University Press, Hong Kong.

Colgan, M. 1987. Coral reef recovery on Guam (Micronesia) after catastrophic predation by Acanthaster planci. Ecology 68:1592-1605.

Connell, J. H., T. E. Hughes, C. C. Wallace, J. E. Tanner, K. E. Harms, and A. M. Kerr. 2004. A long-term study of competition and diversity of corals. Ecological Monographs 74:179-210.

Dai, C.-F. 1988. Coral communities of southern Taiwan. Proceedings of the 6th International Coral Reef Symposium 2:647-652.

Dana, T. F. 1979. Species-numbers relationships in an assemblage of reef building corals: McKean Island, Phoenix Islands. Atoll Research Bulletin 228.

Dikou, A., and R. van Woesik. 2006. Survival under chronic stress from sediment load: spatial patterns of hard coral communities in the southern islands of Singapore. Marine Pollution Bulletin 52:269-280.

Done, T. J. 1985. Effects of two Acanthaster outbreaks on coral community structure - the meaning of devastation. Proceedings of the Fifth International Coral Reef Congress 5:315-320.

Done, T. J., P. K. Dayton, A. E. Dayton, and R. Steger. 1991. Regional and local variability in recovery of shallow coral communities - Moorea, French Polynesia and central Great Barrier Reef. Coral Reefs 9:183-192.

Endean, R., and W. Stablum. 1973. A study of some aspects of the crown-of-thorns starfish (Acanthaster planci) infestations of reefs of Australia's Great Barrier Reef. Atoll Research Bulletin 167:1-76.

Endean, R., and W. Stablum. 1973. The apparent extent of recovery of reefs of Australia's Great Barrier Reef devastated by the crown-of-thorns starfish. Atoll Research Bulletin 168:1-40.

Fisk, D. A., and T. J. Done. 1985. Taxonomic and bathymetric patterns of bleaching in corals, Myrmidon Reef. Proceedings of the Fifth International Coral Reef Congress 6:149-154.

Ginsburg, R.N., ed. 1994. Proceedings of the Colloquium on Global Aspects of Coral Reefs: Health, Hazards and History. University of Miami, Miami.

Gleason, M. G. 1993. Effects of disturbance on coral communities: bleaching in Moorea, French Polynesia. Coral Reefs 12:193-201.

Gomez, E. D., A. C. Alcala, and A. C. San Diego. 1981. Status of Philippine coral reefs -1981. Proceedings of the Fourth International Coral Reef Symposium 1:275-282.

Grigg, R. W. 1994. Effects of sewage discharge, fishing pressure and habitat complexity on coral ecosystems and reef fishes in Hawaii. Marine Ecology Progress Series 103:25-34.

Grigg, R. W. 1995. Coral reefs in an urban embayment in Hawaii: a complex case history controlled by natural and anthropogenic stress. Coral Reefs 14:253-266.

Harriott, V. J., S. D. A. Smith, and P. L. Harrison. 1994. Patterns of coral community structure of subtropical reefs in the Solitary Islands Marine Reserve, Eastern Austraila. Marine Ecology Progress Series 109:67-76.

Hong, G., and A. Sasekumar. 1981. The community structure of the fringing coral reef, Cape Rachado, Malaya. Atoll Research Bulletin 244:1-16.

Hunter, C. L., and C. W. Evans. 1995. Coral reefs in Kaneohe Bay, Hawaii - 2 centuries of western influence and 2 decades of data. Bulletin of Marine Science 57:501-515.

Johnson, C., D. Klumpp, J. Field, and R. Bradbury. 1995. Carbon Flux on Coral-Reefs - Effects of Large Shifts in Community Structure. Marine Ecology-Progress Series 126:123-143.

Jokiel, P. L., and E. K. Brown. 2004. Global warming, regional trends and inshore environmental conditions influence coral bleaching in Hawaii. Global Change Biology 10:1627-1641.

Jones, G. P., M. I. McCormick, M. Srinivasan, and J. V. Eagle. 2004. Coral decline threatens fish biodiversity in marine reserves. Proceedings of the National Academy of Sciences of the United States of America 101:8251-8253.

Kenyon, J. C., P. S. Vroom, K. N. Page, M. J. Dunlap, C. B. Wilkinson, and G. S. Aeby. 2006. Community structure of hermatypic corals at French frigate shoals, Northwestern Hawaiian Islands: Capacity for resistance and resilience to selective stressors. Pacific Science 60:153-175.

Kimrua, T., C.-F. Dai, S. Pae, H. Hui, P. O. Ang, J. G. Je, and C. L. S. Choyce. 2004. Status of coral reefs in East and North Asia: China, Hong Kong, Taiwan, Korea, and Japan. Pages 277-301 *in* C. Wilkinson, editor. Status of coral reefs of the world: 2004. Australian Institute of Marine Science, Townsville.

Lourey, M. J., D. A. J. Ryan, and I. R. Miller. 2000. Rates of decline and recovery of coral cover on reefs impacted by, recovering from and unaffected by crown-of-thorns starfish *Acanthaster planci*: a regional perspective of the Great Barrier Reef. Marine Ecology Progress Series 196:179-186.

Lovell, E., H. Sykes, M. Deiye, L. Wantiez, C. Garrigue, S. Virly, J. Samuelu, A. Solofa, T. Poulasi, K. Pakoa, A. Sabetian, D. Afzal, A. Hughes, and R. Sulu. 2004. Status of coral reefs in the South West Pacific: Fiji, Nauru, New Caledonia, Samoa, Solomon Islands, Tuvalu and Vanuatu. Pages 337-361 *in* C. Wilkinson, editor. Status of coral reefs of the world: 2004. Australian Institute of Marine Science, Townsville.

Maragos, J. E. 1974. Coral communities on a seaward reef slope, Fanning Island. Pacific Science 28:257-278.

Mohamed, M. I. H., and Z. Badaruddin. 1988. Coral reef morphology and ecology of the Malaysian east coast island. Proceedings of the 6th International Coral Reef Symposium 3:349-353.

Moran, P. J., R. H. Bradbury, and R. E. Reichelt. 1985. Mesoscale studies of the Crown-of-Thorns/coral interaction: a case history from the Great Barrier Reef. Proceedings of the Fifth International Coral Reef Congress 5:321-326.

Morton, B. and C.K. Tseng, eds. 1982. The marine flora and fauna of Hong Kong and Southern China. Hong Kong University Press, Hong Kong.

Palaganas, V. P., J. Sy, and P. M. Alino. 1985. Coral communities of the Tubbataha Atolls. Pages 237-242 *in* Proceedings of the Fifth International Coral Reef Congress.

Pearson, R. G. 1974. Recolonization by hermatypic corals of reefs damaged by Acanthaster. Proceedings of the Second International Coral Reef Symposium 2:207-216.

Pearson, R. G. 1981. Recovery and Recolonization of Coral Reefs. Marine Ecology-Progress Series 4:105-122.

Salvat, B. 2002. Status of Southeast and Central Pacific coral reefs 'Polynesia Mana Node': Cook Islands, French Polynesia, Kiribati, Niue, Tokelau, Tonga, Wallis and Futuna. Pages 203-215 *in* C. Wilkinson, editor. Status of coral reefs of the world: 2002. Australian Institute of Marine Science, Townsville.

Sano, M. 2000. Stability of reef fish assemblages: responses to coral recovery after catastrophic predation by Acanthaster planci. Marine Ecology-Progress Series 198:121-130.

Seymour, J. R., N. Patten, D. G. Bourne, and J. G. Mitchell. 2005. Spatial dynamics of virus-like particles and heterotrophic bacteria within a shallow coral reef system. Marine Ecology-Progress Series 288:1-8.

Sulu, R., R. Cumming, L. Wantiez, L. Kumar, A. Mulipola, M. Lober, S. Sauni, T. Poulasi, and K. Pakoa. 2002. Status of coral reefs in the Southwest Pacific to 2002: Fiji, Nauru, New Caledonia, Samoa, Solomon Islands, Tuvalu, and Vanuatu. Pages 181-201 *in* C. Wilkinson, editor. Status of coral reefs of the world: 2002. Australian Institute of Marine Science, Townsville.

Thompson, G. B., and M. Cope. 1982. Estimation of coral abundance by underwater photography. Pages 557-573 *in* B. Morton, editor. Proceedings of the First International Marine Biological Workshop: the marine flora and fauna of Hong Kong and Southern China. Hong Kong University Press, Hong Kong.

Tomascik, T., Suharsono, and A. J. Mah. 1993. Case histories: a historical perspective of the natural and anthropogenic impacts in the Indonesian Archipelago with a focus on the Kepulauan Seribu, Java Sea. Pages 304-310 *in* R. N. Ginsburg, editor. Proceedings of the Colloquium on Global Aspects of Coral Reefs: Health, Hazards, and History. University of Miami, Miami.

Vieux, C., A. Aubanel, J. Axford, Y. Chancerelle, D. A. Fisk, P. Holland, M. Juncker, T. Kirata, M. Kronen, C. Osenberg, B. Pasisi, M. Power, B. Salvat, J. Shima, and V. Vavia. 2004. A century of change in coral reef status in Southeast and Central Pacific: Polynesia Mana Node, Cook Islands, French Polynesia, Kiribati, Niue, Tokelau, Tonga, Wallis and Futuna. Pages 363-380 *in* C. Wilkinson, editor. Status of coral reefs of the world: 2004, Townsville.
