# Supplementary material for: Regional Decline of Coral Cover in the Indo-Pacific: Timing, Extent, and Subregional Comparisons
Source: PLoS One. 2007 Aug 8;2(8):e711. doi: 10.1371/journal.pone.0000711 (PMC1933595; doi:10.1371/journal.pone.0000711)
Supplement: Text S3 — Analysis of potential effects of depth on coral cover estimates (0.05 MB DOC) [file pone.0000711.s003.doc]

**Text S3: Analysis of potential effects of depth on coral cover estimates**

***Changes in survey depth over time:*** A precise depth was reported for 3711 of the 6001 independent reef surveys. The average survey depth was 6.2 m ± 0.05 (mean ± 1 SE). There was a slight change in average survey depth over time, increasing from approximately 5 m in the 1970s to 6 m in the 1990s (linear regression analysis of data pooled across subregions: n = 3711, R^2^adj. = 0.012, p = 0.0001). However, from 1994 to 2004, the relationship reverses and there is a slight but statistically significant decrease in average survey depth of approximately 0.5 m by 2004 (n = 3379, R^2^adj. = 0.003, p = 0.001).

***Variability of survey depth among subregions:*** There was a significant effect of subregion on survey depth (Kruskall-Wallis test on data pooled across years: 3711 observations, df = 9, χ^2^ = 488.9, p < 0.0001). However, the effect was relatively weak and the range in survey depth across all ten subregion was only 4.5 m and the average depth for eight of the ten regions was between 5.8 and 7.6 m. Survey depth also varied slightly among subregions between 1994 and 2004 (3379 observations, df = 9, χ^2^ = 278.1, p < 0.0001) and from 2002-2004 (1117 observations, df = 9, χ^2^ = 107.1, p < 0.0001).

***Effect of depth on cover:*** Survey depth was not significantly related to absolute coral cover either across all years (linear regression analysis of data pooled across subregions: n = 3711, R^2^adj. = 0.0006, p = 0.07), from 1994 to 2004 (n = 3379, R^2^adj. = 0.0004, p = 0.21) or from 2002 to 2004 (n = 1117, R^2^adj. = 0.001, p = 0.19). However, because there were hints of a Depth x Subregion interactive effect on cover (i.e., for seven subregions there was clearly no depth effect, but for three – Great Barrier Reef, Hawaiian Islands, and South Central Pacific – there was a slight positive relationship), we repeated the two main regression analyses including survey depth as a covariate (average annual survey depth for each subregion for the subregional means analysis). Depth was not significantly related to coral cover in either analysis and including depth as a covariate only slightly modified the outcome of the significance tests and parameter estimates for the effect of time (year) on cover (Table S4).
