# Supplementary material for: Regional Decline of Coral Cover in the Indo-Pacific: Timing, Extent, and Subregional Comparisons
Source: PLoS One. 2007 Aug 8;2(8):e711. doi: 10.1371/journal.pone.0000711 (PMC1933595; doi:10.1371/journal.pone.0000711)
Supplement: Text S4 — Potential biases in survey techniques and site selection (0.05 MB DOC) [file pone.0000711.s004.doc]

**Text S4: Potential biases in survey techniques and site selection**

The long-term and regional-scale trends in coral cover we documented cannot be used to describe the state and dynamics of any particular reef. Our results may not even be representative of all ten subregions during the 1970s and early 1980s since relatively few quantitative surveys were performed. Likewise, coral cover on any particular reef or small subset of reefs cannot be extrapolated to larger scales. Given the substantial variability in local reef dynamics, a fairly large population of reefs must be monitored to detect the statistically and ecologically significant signal of regional coral decline.

Our overall approach was to assemble a large regional database so that we could quantify broad-scale and relatively long-term patterns of coral cover across the Indo-Pacific. We wanted a general picture of coral cover across various depths, reef zones and types, subregions, etc. Thus we necessarily included data from several sources. There was some variability in the methodology used to estimate coral cover, although all of the methods we included are known to produce unbiased and relatively accurate estimates of benthic cover. We did not weight data from different sources or exclude studies based on the original purpose of the survey. Dominant reef types and the way reef habitats are characterized vary throughout the Indo-Pacific. Thus we could not make direct geographic comparisons of coral cover while holding factors such as zone, geomorphology, and exposure constant.

Combining data from such a wide variety of individuals and organizations was necessary, but has some obvious drawbacks. Two especially important issues are site selection and the scale of coverage of surveys based on different techniques. Site selection and transect placement are ideally purely random or at least haphazard, but this ideal is not always realized. Either past or present biases in site selection could affect subregional or regional temporal patterns of coral cover. Reefs chosen for some of the single site surveys performed in the early years of the database could have been selected for their high coral coverage, which had a general aesthetic appeal. If many early surveys were biased in this way, our estimates of coral cover decline could be exaggerated. However, a surprising number of the earliest surveys, especially in the early 1970s, surveyed coral cover mainly to document the effect of disturbances [1-3], which could actually have biased site selection within subregions towards low cover reefs.

**Manta tow data:** We only used coral cover data from manta tow surveys performed by the Australian Institute of Marine Science’s (AIMS) Long-Term Monitoring Program. Comparisons of AIMS manta tow data with video transect data from the same reefs indicate that estimates of coral cover derived from AIMS manta tows are quite accurate and comparable to smaller scale and more precise survey techniques [4]. However, the manta tows of the GBR used in our database cover much more reef area and a far greater range of reef habitats than the video transect surveys of permanently marked sites, which are situated on the relatively high cover northeastern outer reef slopes [4,5]. As a result, coral cover measured by AIMS on the permanent reef slope video transects is typically higher than their measurements from the more extensive manta tows [5]. It is important to note that this is due to the greater sampling grain and habitat coverage of the AIMS manta tows rather than a bias of the technique.

The AIMS manta tow survey data are more analogous to cover measurements in the other nine subregions than the AIMS video transects because we included surveys from a variety of reef zones and depths. Some of the surveys performed in the other nine subregions were on high cover fore reef slopes, but many were also in habitats with typically lower coral cover such as exposed, shallow zones and back reef environments.

**Reef Check data:** It is possible that the estimated recent and current coral cover in subregions with substantial Reef Check data was inflated by the Reef Check site selection protocol. Reef Check instructs team leaders to survey the healthiest local reefs: “Teams were instructed to survey outer slopes on exposed reefs that were considered to be the ‘best’ sites in their area – those believed to be least affected by human activities and having the highest percentage of the seabed covered by living hard coral" [6]. From 1997 to 2004, 39% to 75% of the total annual coral cover estimates were based on Reef Check surveys. Therefore we may have underestimated the rate and extent of regional coral decline.

Our estimate of current coral cover is possibly higher than the true average because 259 of the 390 surveys performed in 2003 were based on the Reef Check methodology. Additionally, this could have confounded comparisons with subregions in which relatively few Reef Check surveys were performed. The Reef Check cover estimate for 2003 of 21.1% ± 0.62 (n = 259) might be considered the average of the healthiest reefs in the subregions surveyed extensively by Reef Check (mainly the southwestern Pacific, Indonesia, mainland Asia, and the Philippines). However, the 2003 Reef Check cover mean is slightly *lower* (t = 2.0, p = 0.04) than the non-Reef Check estimate of 24.1 ± 1.6 (n = 133). In fact, our analysis of the effect of the data source on measured coral cover suggests that the site selection bias in the Reef Check methodology might not overestimate subregional or regional coral cover.

A robust quantitative comparison between Reef Check and non-Reef Check cover estimates is not possible because few subregions have been adequately surveyed in a given year using both methods. But we cautiously compared the annual mean cover estimates for subregions surveyed by both Reef Check and non-Reef Check teams between 1997 and 2004 with a paired t test: the Reef Check mean (20.3 ± 0.8) was significantly lower than the non-Reef Check mean (32.1 ± 2.3) across the 21 subregion/year pairs for which data are available (t = 4.5, p = 0.0002). These results suggest that the Reef Check site selection protocol might not bias estimates of subregional coral cover by preferentially surveying the highest cover reefs. One reason for the lack of a significant bias is that there are very few remaining high cover reefs that could appreciably influence subregional averages (i.e., because there is little difference between the ‘healthiest’ local reef and the average local reef). It is also possible that Reef Check volunteer teams lack the resources to adequately pre-survey local areas to a degree that would enable them to effectively identify non-representative high cover reefs.

**References**

1. Chesher RH (1969) Destruction of Pacific corals by the sea star Acanthaster planci. Science 165: 280-283.

2. Endean R, Stablum W (1973) The apparent extent of recovery of reefs of Australia's Great Barrier Reef devastated by the crown-of-thorns starfish. Atoll Research Bulletin 168: 1-41.

3. Grigg RW (1994) Effects of sewage discharge, fishing pressure and habitat complexity on coral ecosystems and reef fishes in Hawaii. Marine Ecology Progress Series 103: 25-34.

4. Miller I, Müller R (1999) Validity and reproducibility of benthic cover estimates made during broadscale surveys of coral reefs by manta tow. Coral Reefs 18: 353-356.

5. Sweatman H, Abdo D, Burgess S, Cheal A, Coleman G, et al. (2004) Long-term monitoring of the Great Barrier Reef: status report number 6. Townsville: Australian Institute of Marine Science. 254 p.

6. Hodgson G (1999) A global assessment of human effects on coral reefs. Marine Pollution Bulletin 38: 345-355.
