# Supplementary material for: Regional Decline of Coral Cover in the Indo-Pacific: Timing, Extent, and Subregional Comparisons
Source: PLoS One. 2007 Aug 8;2(8):e711. doi: 10.1371/journal.pone.0000711 (PMC1933595; doi:10.1371/journal.pone.0000711)
Supplement: Table S1 — The number of surveyed reefs in the ten Indo-Pacific subregions. (0.05 MB DOC) [file pone.0000711.s005.doc]

**Table S1.** The number of surveyed reefs in the ten Indo-Pacific subregions.

| **Subregion** | **1960-1983** | **1984-1996** | **1997-2004** | **Total** |
| --- | --- | --- | --- | --- |
| East Indonesia & PNG | 1 | 200 | 488 | 689 |
| Great Barrier Reef | 104 | 817 | 981 | 1902 |
| Hawaiian Islands | 17 | 53 | 274 | 344 |
| Mainland Asia | 68 | 342 | 332 | 742 |
| Philippines | 286 | 646 | 178 | 1110 |
| Southwestern Pacific | 0 | 8 | 225 | 233 |
| South Pacific | 2 | 23 | 56 | 81 |
| Taiwan & Japan | 8 | 49 | 161 | 218 |
| West Indonesia | 23 | 298 | 221 | 542 |
| Western Pacific | 16 | 0 | 124 | 140 |
| All subregions | 525 | 2436 | 3040 | 6001 |
