# Supplementary material for: Regional Decline of Coral Cover in the Indo-Pacific: Timing, Extent, and Subregional Comparisons
Source: PLoS One. 2007 Aug 8;2(8):e711. doi: 10.1371/journal.pone.0000711 (PMC1933595; doi:10.1371/journal.pone.0000711)
Supplement: Table S2 — Characteristics of the eight basic sources of coral cover data. (0.05 MB DOC) [file pone.0000711.s006.doc]

**Table S2. Characteristics of the eight basic sources of coral cover data.**

| **Source** | **Subregions** | **Surveys** | **Reefs** | **Years** |
| --- | --- | --- | --- | --- |
| Australian Institute of Marine Science (AIMS) | GBR | 1677 | 185 | 1986-2004 |
| Gray literature including conference proceedings and edited books | EIPNG, MA, PHI, SWP, SP, TJ, WI, WP | 457 | 205 | 1971-2004 |
| Hawaii Coral Reef Assessment and Monitoring Program (CRAMP) | HI | 238 | 36 | 1973-2002 |
| Marine Science Institute, University of the Philippines, Diliman, Quezon City, Philippines | PHI | 140 | 140 | 1991, 1995,1997 |
| National Oceanic and Atmospheric Administration (NOAA) | HI | 26 | 26 | 2002 |
| Peer reviewed literature | EIPNG, GBR, MA, HI, PHI, SP, TJ, WP | 300 | 147 | 1968-2003 |
| ReefBase | EIPNG, MA, PHI, SWP, TJ, WI | 1292 | 839 | 1971-2000 |
| Reef Check | All | 1501 | 799 | 1997-2004 |
| The Nature Conservancy | EIPNG | 370 | 346 | 1996, 1998 |

Subregion codes: East Indonesia & PNG (EIPNG), Great Barrier Reef (GBR), Hawaiian Islands (HI), Mainland Asia (MA), Philippines (PHI), Southwestern Pacific (SWP), South Pacific (SP),Taiwan & Japan (TJ), West Indonesia (WI), Western Pacific (WP)
