# Supplementary material for: Regional Decline of Coral Cover in the Indo-Pacific: Timing, Extent, and Subregional Comparisons
Source: PLoS One. 2007 Aug 8;2(8):e711. doi: 10.1371/journal.pone.0000711 (PMC1933595; doi:10.1371/journal.pone.0000711)
Supplement: Table S3 — Number of monitoring sites in the ten Indo-Pacific subregions during each of three periods (note most monitoring sites were surveyed for more than one period). (0.05 MB DOC) [file pone.0000711.s007.doc]

**Table S3.** Number of monitoring sites in the ten Indo-Pacific subregions during each of three periods (note most monitoring sites were surveyed for more than one period).

| **Subregion** | **1970-1983** | **1984-1996** | **1997-2004** | **Total** |
| --- | --- | --- | --- | --- |
| East Indonesia & PNG | 0 | 25 | 44 | 45 |
| Great Barrier Reef | 21 | 185 | 195 | 213 |
| Hawaiian Islands | 5 | 6 | 38 | 40 |
| Mainland Asia | 26 | 45 | 56 | 99 |
| Philippines | 50 | 110 | 43 | 136 |
| Southwestern Pacific | 0 | 0 | 38 | 38 |
| South Pacific | 1 | 7 | 11 | 12 |
| Taiwan & Japan | 1 | 3 | 20 | 21 |
| West Indonesia | 5 | 14 | 6 | 21 |
| Western Pacific | 1 | 1 | 25 | 26 |
| All subregions | 110 | 396 | 476 | 651 |
