# Supplementary material for: Regional Decline of Coral Cover in the Indo-Pacific: Timing, Extent, and Subregional Comparisons
Source: PLoS One. 2007 Aug 8;2(8):e711. doi: 10.1371/journal.pone.0000711 (PMC1933595; doi:10.1371/journal.pone.0000711)
Supplement: Table S4 — Results of linear repeated measures regression analyses on the relationship between coral cover and time in the Indo-Pacific. Unlike the results presented in Table 1, these analyses include survey depth as a covariate. The effect of depth was non-significant (Subregional analysis p = 0.90, Monitoring sites p = 0.60). Results presented in the table are for the time effect. n = total number of observations (0.04 MB DOC) [file pone.0000711.s008.doc]

**Table S4.** Results of linear repeated measures regression analyses on the relationship between coral cover and time in the Indo-Pacific. Unlike the results presented in Table 1, these analyses include survey depth as a covariate. The effect of depth was non-significant (Subregional analysis p = 0.90, Monitoring sites p = 0.60). Results presented in the table are for the time effect. n = total number of observations

| **Analysis** | **n** | **df** | **p** | **R^2^** | **Slope** (95% CI) |
| --- | --- | --- | --- | --- | --- |
| Subregional means  (1968-2004) | 152 | 2, 9 | < 0.00001 | 0.13 | -0.47 (-0.66, -0.28) |
| Monitoring sites  (1970-2004) | 2807 | 2, 462 | 0.005 | 0.01 | -0.25 (-0.43, -0.08) |
